# Draft genome sequence of *Lampropedia cohaerens* strain CT6^T^ isolated from arsenic rich microbial mats of a Himalayan hot water spring

**DOI:** 10.1186/s40793-016-0179-1

**Published:** 2016-09-07

**Authors:** Charu Tripathi, Nitish K. Mahato, Pooja Rani, Yogendra Singh, Komal Kamra, Rup Lal

**Affiliations:** 1 Molecular Biology Laboratory, Department of Zoology, University of Delhi, Delhi, 110007 India; 2 Department of Zoology, University of Delhi, Delhi, 110007 India; 3 Ciliate Biology Laboratory, SGTB Khalsa College, University of Delhi, Delhi, 110007 India

**Keywords:** *Lampropedia cohaerens*, Hot spring, Biofilm, Mineral phosphate solubilisation, Arsenic tolerance, Pyrroloquinoline-quinone

## Abstract

*Lampropedia cohaerens* strain CT6^T^, a non-motile, aerobic and coccoid strain was isolated from arsenic rich microbial mats (temperature ~45 °C) of a hot water spring located atop the Himalayan ranges at Manikaran, India. The present study reports the first genome sequence of type strain CT6^T^ of genus *Lampropedia cohaerens*. Sequencing data was generated using the Illumina HiSeq 2000 platform and assembled with ABySS v 1.3.5. The 3,158,922 bp genome was assembled into 41 contigs with a mean GC content of 63.5 % and 2823 coding sequences. Strain CT6^T^ was found to harbour genes involved in both the Entner-Duodoroff pathway and non-phosphorylated ED pathway. Strain CT6^T^ also contained genes responsible for imparting resistance to arsenic, copper, cobalt, zinc, cadmium and magnesium, providing survival advantages at a thermal location. Additionally, the presence of genes associated with biofilm formation, pyrroloquinoline-quinone production, isoquinoline degradation and mineral phosphate solubilisation in the genome demonstrate the diverse genetic potential for survival at stressed niches.

## Introduction

The genus *Lampropedia*, a member of the family *Comamonadaceae* [1] was established by Schroeter in 1886 [2] with the description of square, tablet forming cells of *Lampropedia hyalina*. Henceforth, strains of the same species, *L. hyalina* have been isolated from pond water [2], liquid manure of a dairy farm yard [3], fistulated heifer [4] and activated sludge [5]. *L. hyalina* was isolated from activated sludge, and was tested for its phosphate removal capabilities and was classified as belonging to the functional group of polyphosphate accumulating microorganisms [5]. Another species, *L. cohaerens* strain CT6^T^ [6] was isolated from arsenic rich microbial mats of a Himalayan hot water spring from Manikaran, India as a continuation to our efforts to explore the culturable [7–10] and unculturable [11] diversity at the Himalayan hot spring to understand the role played by niche specific genetic determinants in shaping the genomes of organisms inhabiting this stressed niche. *L. cohaerens*, a biofilm forming and arsenic tolerating bacterium [6], showed limited carbohydrate assimilation potential but could utilize some organic acids. Currently, the genus *Lampropedia* is represented by three species, *L. hyalina*ATCC 11041^T^ [12], “*L. puyangensis* 2-bin^T^” (not validly published) [13] and *L. cohaerens* CT6^T^ [6], leading to the description of the genus being emended [6], however, the genomic potential of this small group remains unresolved. The genome of strain CT6^T^, which is the type strain for *Lampropedia cohaerens* was sequenced in order to supplement the phenotypic taxonomical observations with genetic data and obtain genomic insights into heavy metal resistance and metabolic potential of gene complements of this microbial mat dweller. Here, we describe the summary classification, properties, genome sequencing, assembly and annotation of *L. cohaerens* CT6^T^ (DSM 100029^T^=KCTC 42939^T^).

## Organism information

### Classification and features

**Fig. 1:**
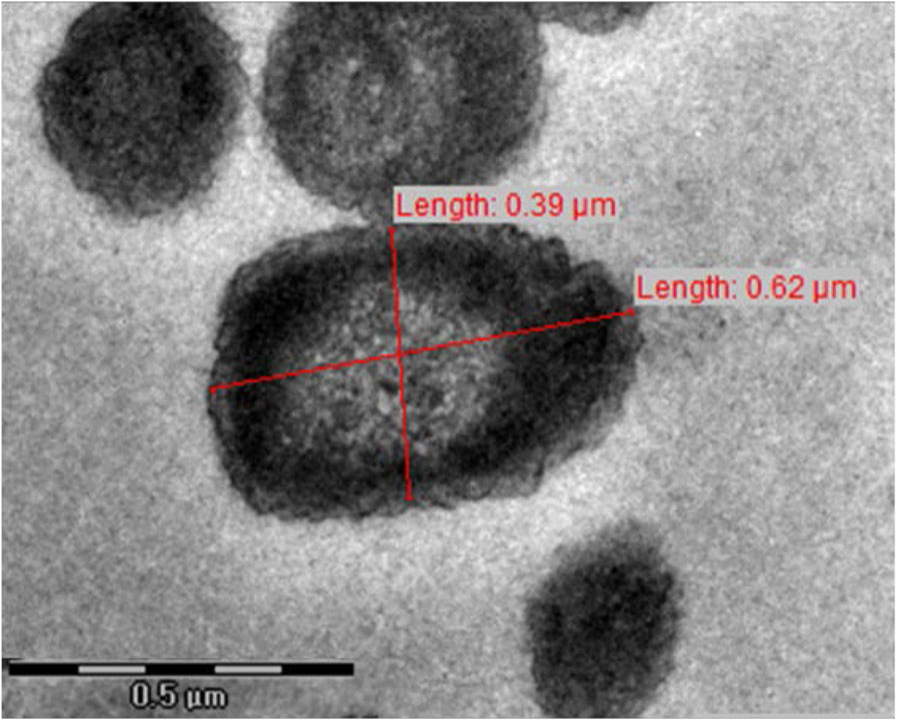
TEM of *Lampropedia cohaerens* strain CT6^T^ cells. Length of bar = 0.5 μm

**Fig. 2:**
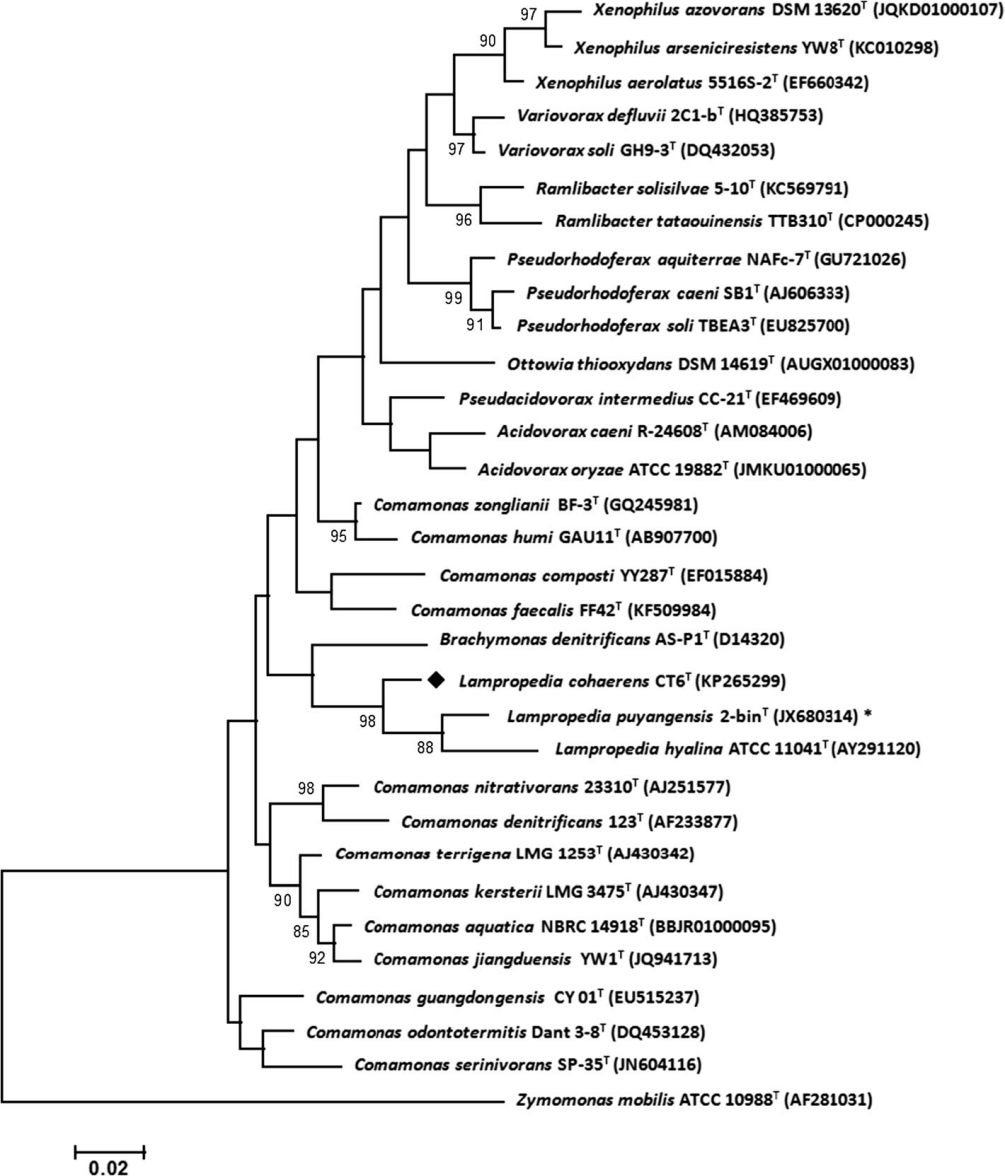
Maximum-Likelihood phylogenetic tree based on 16S rRNA gene sequences of *L. cohaerens* strain CT6^T^ and its nearest phylogenetic neighbours based on blast-n similarity. All phylogenetic neighbours belong to the family *Comamonadaceae*. The tree was computed using the Jukes and Cantor model. Bootstrap values (>70 %) calculated for 1000 subsets are shown at branch points. Bar 2 substitutions per 100 nucleotide positions. *Not validly published

*L. cohaerens* was characterized by using a polyphasic approach with the integration of genotypic, phenotypic and chemotaxonomic methods [6]. This Gram-stain-negative, aerobic bacterial strain, forms white, smooth colonies with irregular margins on LB agar [6]. Transmission electron microscopy (TEM) revealed coccoid, unflagellated cells approximately 0.62 μm × 0.39 μm in dimension (Fig. 1). Summary characteristics are mentioned in Table 1. The slightly thermophilic and arsenic tolerant *L. cohaerens* strain CT6 can tolerate temperature in the range 20–55 °C and can tolerate arsenic trioxide up to 80 parts per billion [6]. The NaCl tolerance for strain CT6^T^ was tested as 1–3 % (w/v) and pH range as 6–9. Biofilm formation is observed in LB media, inspiring its etymology. *L. cohaerens* showed closest phylogenetic similarity to “*L. puyangensis* 2-bin^T^” (96.4 %) and *L. hyalina*ATCC 11041^T^ (95.4 %) on the basis of 16S rRNA gene sequences. A maximum-likelihood [14] phylogenetic tree based on Jukes-Cantor [15] model using MEGA version 6 [16] constructed with closely related members of family *Comamonadaceae* on the basis of Blast-n [17] of 16S rRNA gene placed strain CT6^T^ along with the members of genus *Lampropedia* with bootstrap [18] confidence value of 98 % (Fig. 2). Positive biochemical tests included the hydrolysis of tween 20, tween 80 and starch and utilization of capric acid, malic acid, citric acid, xanthine and hypoxanthine [6]. Catalase test was positive whereas oxidase test was negative [6]. The most prominent fatty acid methyl esters were C_16:0_, summed feature 8 (C_18:1_*ω7c*/C_18:1_*ω6c*), C_14:0_, C_19:0_*ω8c* cyclo and summed feature 3 (C_16:1_*ω7c*/C_16:1_*ω6c*) [6]. The major polar lipids detected in strain CT6^T^ were phosphatidylethanolamine, phosphatidylglycerol and a glycolipid [6]. Strain CT6^T^ demonstrated the presence of putrescine, 2-hydroxyputrescine and spermidine as the major polyamines and ubiquinone-8 as the major quinone [6].

**Table 1:** Classification and general features of *Lampropedia cohaerens* CT6^T^ [39, 40]

MIGS Id	Property	Term	Evidence code^a^
	Classification	Domain *Bacteria*	TAS [41]
		Phylum *Proteobacteria*	TAS [42, 43]
		Class *Betaproteobacteria*	TAS [44, 45]
		Order *Burkholderiales*	TAS [44, 46]
		Family *Comamonadaceae*	TAS [1]
		Genus *Lampropedia*	TAS [2, 47]
		Species *cohaerens*	TAS [6]
		Type strain: Strain CT6^T^ (Accession: DSM 100029^T^)	TAS [6]
	Gram stain	Negative	TAS [6]
	Cell-shape	Coccoid	TAS [6]
	Motility	Non-motile	TAS [6]
	Sporulation	Not reported	NAS
	Temperature range	20–55 °C	TAS [6]
	Optimum temperature	37 °C	TAS [6]
	pH range; Optimum	6–9	TAS [6]
	Carbon source	Capric acid, Malic acid, Citric acid	TAS [6]
MIGS-6	Habitat	Microbial mat	TAS [6]
MIGS-6.3	Salinity	1–3 % NaCl (w/v)	TAS [6]
MIGS-22	Oxygen requirement	Aerobic	TAS [6]
MIGS-15	Biotic relationship	Free-living	NAS
MIGS-14	Pathogenicity	Non-pathogenic	NAS
MIGS-4	Geographic Location	India	TAS [6]
MIGS-5	Sample collection	2014	IDA
MIGS-4.1	Latitude	31.378473	IDA
MIGS-4.2	Longitude	77.406945	IDA
MIGS-4.4	Altitude	1700 m	IDA

^a^Evidence codes - *IDA* Inferred from Direct Assay, *TAS* Traceable Author Statement (i.e., a direct report exists in the literature), *NAS* Non-traceable Author Statement (i.e., not directly observed for the living, isolated sample, but based on a generally accepted property for the species, or anecdotal evidence). These evidence codes are from the Gene Ontology project [48]

## Genome sequencing information

### Genome project history

Whole genome sequencing was performed at Beijing Genomics Institute Technology Solutions, Hong Kong, China using the Illumina HiSeq 2000 technology. Sequencing was done using 500 bp and 2 kbp paired end libraries. Raw data was generated within a duration of 3 months. *De-novo* assembly was performed in-house at the University of Delhi. The draft genome sequence was submitted to NCBI under the accession number LBNQ00000000 (version 1 LBNQ01000000). The sequences were also submitted to IMG-JGI portal under GOLD Analysis Project ID Ga0079366. Sequence project information in compliance with MIGS version 2.0 is given in Table 2.

**Table 2:** Project information

MIGS ID	Property	Term
MIGS 31	Finishing quality	Improved-High-Quality Draft
MIGS 28	Libraries used	500-bp and 2-kbp paired-end library
MIGS 29	Sequencing platform	Illumina HiSeq 2000
MIGS 31.2	Fold coverage	>10×
MIGS 30	Assemblers	ABySS v 1.3.5
MIGS 32	Gene calling method	Prodigal 1.4
	Locus Tag	AAV94
	Genbank ID	LBNQ00000000
	Genbank Date of Release	May 8, 2015
	GOLD ID	Ga0079366
	BIOPROJECT	PRJNA282900
MIGS 13	Source material identifier	DSM 100029, KCTC 42939
	Project relevance	Heavy metal tolerant, biofilm forming bacterium

### Growth conditions and genomic DNA preparation

Genomic DNA was isolated from a 25 ml culture grown in LB medium incubated at 37 °C. Mid-logarithmic phase culture (O.D. 0.6) was harvested and cells were lysed in TE25S buffer (25 mM Tris-HCl pH 8.0, 25 mM EDTA, 0.3 M sucrose, 1.0 mg/ml lysozyme), followed by removal of proteins by 1.0 % SDS and 1.0 mg/ml proteinase-K at 55 °C. This was followed by DNA purification steps using Phenol : Chloroform : Isoamyl alcohol (25 : 24 : 1) and Chloroform : Isoamyl alcohol (24 : 1). DNA was precipitated using 0.6 volume of Isopropanol. After washing with 70 % ethanol, DNA was dissolved in 5 mM Tris-EDTA. Sample concentration was estimated as 347.1 ng/μl by microplate reader and integrity was checked using agarose gel electrophoresis prior to sequencing. Purity ratios 260/280 and 260/230 were 1.89 and 1.91 respectively.

### Genome sequencing and assembly

Genomic DNA was sequenced using 500 bp and 2 kbp paired-end libraries. Raw read filtering and removal of adapters were carried out at the BGI Technology Solutions Co. Limited, China. A total of 7.5 Gb raw data was generated with 33,961,144 clean reads encompassing a total of 3,056,502,960 clean bases. *De-novo* assembly of raw reads using ABySS version 1.3.5 [19] generated 41 contigs greater than 500 bp at k-mer 51 with n50 value of 165,853. Assembly validation was done by aligning raw reads onto finished contigs using Burrows Wheeler Aligner version 0.7.9a [20] followed by visual inspection using Tablet version 1.14.04.10 [21]. The final draft was assembled into 41 contigs with a mean contig size of 77,047 bp. The assembled genome had 3,158,922 bases with 63.5 % G+C content.

### Genome annotation

For initial annotations, sequences were submitted to the NCBI Prokaryotic Genomes Annotation Pipeline. Additionally, the sequences were uploaded on Integrated Microbial Genomes pipeline [22] under the umbrella of Joint Genome Institute [22]. Coding sequence prediction was performed using Prodigal V2.6.2 [23]. rRNA operons were predicted using RNAmmer version 1.2 [24]. tRNAs and tmRNAs were predicted using ARAGORN [25]. Phage Search Tool [26] was used to find phages in the genome. CRISPRs were found online by CRISPR finder online server [27]. For prediction of signal peptides and transmembrane domains, SignalP 4.1 server [28] and TMHMM server v. 2.0 [29] were used respectively. COG category assignment and Pfam domain predictions were done using WebMGA server [30].

## Genome properties

**Fig. 3:**
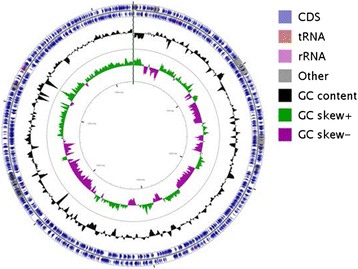
A graphical circular map of the genome performed with CGview comparison tool [49]. From outside to centre, ring 1 and 2 show protein coding genes on both the forward and reverse strand; ring 3 shows G+C% content plot, and ring 4 shows GC skew

The final draft genome consists of 41 contigs with a total of 3,158,922 bp and a G+C mol% of 63.5. A total of 2909 coding sequences were predicted accounting for a coding density of 88.92 %. Out of the total coding sequences, 83.84 % were assigned functions. Protein coding genes were 2823 and comprised 97.04 % of the total; RNA coding genes were 86 in number and 56 tRNAs were detected. Five rRNA operons were predicted with complete 5S-16S-23S rRNA genes (Fig. 3). Three confirmed CRISPRs were detected, one on contig 13 and two on contig 33. Two incomplete phages were also detected having a phage integrase and an *attR* site for integration. Pfam domains were detected for 2539 genes, 238 genes were found to code for proteins harbouring signal peptides and 665 genes with transmembrane domains (Table 3). Out of the total genes, 2713 (92.09 %) were assigned to COG categories. COG category assignment placed majority of genes to general function prediction only (10.62 %), amino acid transport and metabolism (10.31 %), inorganic ion transport and metabolism (6.92 %) and energy production and conversion (6.21). 6.24 % genes were placed in the function unknown category, whereas 7.91 % genes were not placed into the COGs (Table 4).

**Table 3:** Genome statistics

Attribute	Genome (total) Value	% of total^a^
Genome size (bp)	3,158,922	100.00
DNA coding (bp)	2,808,764	88.92
DNA G+C (bp)	2,004,739	63.46
DNA scaffolds	41	100.00
Total genes	2909	100.00
Protein coding genes	2823	97.04
RNA genes	86	2.96
Pseudo genes	0	0.00
Genes in internal clusters	302	10.38
Genes with function prediction	2439	83.84
Genes assigned to COGs	2713	92.09
Genes with Pfam domains	2539	87.28
Genes with signal pedtides	238	8.18
Genes with transmembrane helices	665	22.86
CRISPR repeats	3	100.00

^a^The total is based on either the size of the genome in base pairs or the total number of protein coding genes predicted in the annotated draft genome

**Table 4:** Number of genes associated with general COG functional categories

Code	Value	%age	COG category
J	169	5.70	Translation, ribosomal structure and biogenesis
A	1	0.03	RNA processing and modification
K	172	5.84	Transcription
L	131	4.45	Replication, recombination and repair
B	3	0.10	Chromatin structure and dynamics
D	27	0.92	Cell cycle control, cell division, chromosome partitioning
V	37	1.25	Defense mechanisms
T	119	4.04	Signal transduction mechanisms
M	163	5.53	Cell wall/membrane/envelope biogenesis
N	39	1.32	Cell motility
U	83	2.82	Intracellular trafficking, secretion, and vesicular transport
O	106	3.59	Posttranscriptional modification, protein turnover, chaperones
C	183	6.21	Energy production and conversion
G	111	3.76	Carbohydrate transport and metabolism
E	304	10.31	Amino acid transport and metabolism
F	71	2.41	Nucleotide transport and metabolism
H	111	3.76	Coenzyme transport and metabolism
I	110	3.73	Lipid transport and metabolism
P	204	6.92	Inorganic ion transport and metabolism
Q	72	2.44	Secondary metabolite biosynthesis, transport and catabolism
R	313	10.62	General function prediction only
S	184	6.24	Function unknown
-	233	7.91	Not in COGS

## Insights from the genome sequence

Consistent with the limited metabolic potential of *L. cohaerens*, the genome sequence was found to lack hexokinase and glucokinase, key enzymes involved in glycolysis. Additionally, the lack of pentose phosphate pathway genes glucose-6-phosphate 1-dehydrogenase and 6-phosphogluconolactonase are responsible for the organism’s inability to utilize carbohydrates. However, genes involved in Entner-Doudoroff pathway and non-phosphorylated ED pathways were identified. nED pathway enzyme D-gluconate dehydratase (EC 4.2.1.39) which brings about the conversion of D-gluconate to 2-keto-3-deoxy-D-gluconate [31] was identified, along with conventional ED pathway enzyme 2-keto-3-deoxy-6-phosphogluconate aldolase (EC 4.1.2.14) which brings about the conversion of KDPG (generated after the first step in ED pathway) to pyruvate and glyceraldehyde-3-phosphate [32]. Although *L. cohaerens* possesses enzymes involved in both the ED and nED pathway, the link between the two could not be established as the enzyme KDG kinase which brings about the conversion of KDG to KDPG could not be identified.

*L. cohaerens* CT6^T^ was isolated from hot spring microbial mats, known to be rich in heavy metal sulfides. Microbiota present at hot springs have developed resistance mechanisms to withstand and survive high heavy metal concentrations. Consequently, *L. cohaerens* demonstrated a repertoire of heavy metal resistance genes. Among genes imparting resistance against arsenic, arsenate reductase genes *arsC* (AAV94_10615), arsenic resistance genes *arsH* (AAV94_10620)*,* arsenic transporter *ACR3* (AAV94_10610) and transcriptional regulator *arsR* (AAV94_10600, AAV94_10605) were found. Two arsenic resistance clusters were found on contig 33 harbouring two copies of *arsR,* a copy of *ACR3,* and a copy of arsenate reductase *arsC.* In one of the clusters, an additional gene *arsH,* coding for arsenical resistance protein was found. Additionally, a gene *arsB* coding for arsenic efflux pump protein was identified. Among heavy metals, copper, a trace mineral element is taken up by living cells to get incorporated into a number of enzymes, particularly cytochrome oxidases; however, in excess it becomes toxic to the cells. Copper resistance mechanisms in bacteria involve the *cus* system, the *cue* system and the *pco* system [33]. Excess copper is removed either by efflux of the cations or by periplasmic detoxification. *Cue* system genes *copA,* an ion translocating ATPase; *cueO* [34]*,* a perplasmic multicopper oxidase and *cueR,* a copper response metalloregulatory protein which acts as the regulator of both *copA* and *cueO* [35] were identified. *Cus* system genes *cusA* and *cusB* both coding for cation efflux proteins are harboured by *L. cohaerens**.* Additionally, *pco* system genes, *copC* and *copD* were present in its genome. Genes imparting resistance to other heavy metals including cobalt, zinc and cadmium efflux system genes *czcA, czcD,czcB* and *czcC* which code for outer membrane transporter efflux proteins were identified. Magnesium and cobalt transport protein encoding genes *corA* and *corC* were identified. Transcriptional regulators of *merR* family were found in six copies. *MerR* transcriptional factors are known to be regulators of various environmental stimuli, particularly, high concentrations of heavy metals and oxidative stress [36].

The genetics of biofilm formation in bacteria is a complex process and is dependent on the modulation of expression of a number of genes, mainly those involved in adhesion and autoregulation [37]. The PGA operon is comprised of genes coding for the synthesis of a secreted polysaccharide poly-β-1,6-N-acetyl-D-glucosamine responsible for cell-cell and cell-surface adhesion in biofilms. Strain CT6^T^ demonstrated biofilm formation in vitro, the genes responsible for which were found in its genome. The PGA operon genes *pgaA* - biofilm secretion outer membrane secretion, *pgaB* - biofilm PGA synthesis deacetylase and *pgaC* - biofilm PGA synthesis N-glucosyltransferase were found to be harboured as a single operon in the genome.

Members of the family *Comamonadaceae* have been shown to possess a mineral phosphate solubilisation phenotype. Genes associated with the MPS phenotype include a glucose dehydrogenase and a pyrroloquinoline-quinone synthase system. PQQ is a cofactor for glucose dehydrogenase. PQQ, a small molecule that serves as a redox cofactor in several enzymes has been found to be produced by *Pseudomoas fluorescens,**Enterobacter intermedium* and many other bacteria. PQQ production has been shown to be involved in plant growth promoting effects in soil dwelling bacteria. Additionally, PQQ production has been associated with higher tolerance to radiation and free oxygen radicals, thus bringing to light its free radical scavenging role in bacteria [38]. PQQ dependent enzymes like GDH play a role in the availability of insoluble phosphates to plants, thus contributing to their mineral phosphate solubilisation phenotype. The MPS phenotype contributes significantly to the mineralization of phosphates, playing a key role in geochemical cycling of the element. Consequently, three copies of PQQ dependent glucose dehydrogenase gene were found. PQQ synthase genes *pqqB, D, E* were also found. Further, genes coding for isoquinoline 1-oxidoreductase α and β subunit corresponding to the isoquinoline degradation system were found. Isoquinoline 1-oxidoreductase catabolizes the first step in the hydroxylation of isoquinoline, a N-heterocyclic compound which is commonly associated with coal gasification, shale oil, coal tar, crude oil contaminated sites.

## Conclusions

The genome of *L. cohaerens* strain CT6^T^*,* a biofilm forming and arsenic tolerating bacterium was found to harbour the genes necessary for arsenic tolerance and biofilm formation. Genes related with the transport and efflux of copper, cobalt, zinc and cadmium were identified. Limited metabolic potential was attributed to lack of key glycolysis and pentose phosphate pathway genes. A metabolically unique combination of genes involving both ED pathway and the nED pathway was encountered. Phylloquinoline-quinone synthetic genes were identified along with PQQ requiring glucose dehydrogenase. This was consistent with the phosphate removal phenotype of *Lampropedia* from sewage slugde samples [5]. *L. cohaerens*, which harbours MPS phenotype imparting genes, can be considered to belong to the group of MPS bacteria which are used to enhance the fertility of soil by ensuring availability of trapped phosphates to plants. The presence of isoquinoline degrading genes may be employed for removal of oil contaminations. Further experiments can be performed to link the genetic determinants of *L. cohaerens* with its actual functional potential. The genetic repertoire of *L. cohaerens* points towards survival capabilities at diverse stressed niches. The genes harboured by *L. cohaerens* enable the organism to survive at heavy metal rich microbial mats of hot spring. Biofilm formation may be considered as a niche specialised strategy adapted to survive the hot spring waters forming microbial mats. The diverse survival instincts are reflected in the genome by the presence of genes for a PQQ synthase system and PQQ-dependent glucose dehydrogenases. Isoquinoline degradation genes provide a supplemental benefit for survival at oil contaminated sites. Further, the presence of isoquinoline-degradation genes makes *L. cohaerens* a potential candidate for bioremediation of oil contaminated sites.

## Abbreviations

BGI: Beijing Genomics Institute
COG: Cluster of orthologous groups
ED: Entner-Duodoroff pathway
GDH: Glucose dehydrogenase
IMG: Integrated microbial genomes
KDG: 2-keto-3-deoxy-D-gluconate
KDPG: 2-keto-3-deoxy-6-phosphogluconate
MPS: Mineral phosphate solubilisation
nED: Non-phosphorylated ED pathway
PQQ: Pyrroloquinoline-quinone
